# Nonlinear control of switchable wavelength-selective absorption in a one-dimensional photonic crystal including ultrathin phase transition material-vanadium dioxide

**DOI:** 10.1038/s41598-022-14486-2

**Published:** 2022-06-23

**Authors:** Ziba Saleki

**Affiliations:** Department of Opto-Electronics and Information Engineering, College of Precision Instruments and Opto-Electronics Engineering, Tianjin University, Tianjin, 300072 People’s Republic of China

**Keywords:** Materials science, Nanoscience and technology, Optics and photonics, Physics

## Abstract

Based on the transfer matrix theory, I realize a nearly perfect wavelength-selective absorption of near-IR waves in a one-dimensional defective photonic crystal, $(AB)^ND(BA)^M$, containing a vanadium dioxide (VO$_2$) phase transition layer as a defect. Firstly, the effect of the period numbers, *N* and *M*, on the absorption spectrum is studied to achieve a perfect absorption peak. It is shown that optimal period numbers of the structure to maximize the absorption peak are N = 7 and M = 16. Our results also indicate that a narrow-band, almost perfect absorption is achieved due to the symmetry of the structure with respect to VO$_2$. Also, the absorption amount of the considered structure is about 50 times larger than that of a free-standing VO$_2$. Furthermore, the absorption peak value and resonant wavelength can be continuously tuned while VO$_2$ transits from semiconductor to metal phase at 340 K temperature. In addition, how different parameters such as the polarization and incident angle affect the absorption spectra is discussed. Finally, the nonlinear absorption spectra of the structure are graphically demonstrated beside the linear case. The current system can be applied in designing practical tunable optical devices such as IR sensors, limiters, and switches.

## Introduction

As the quest for active optical systems arouses a growing interest, substances with peculiar and exploitable dynamic control characteristics have become paramount to enable novel developments in practical applications including microfluidic sensors, optical switching devices, thermal emitters, filters, and modulators^1–9^. Of particular burgeoning interest is the study of the absorption behavior in both science and engineering communities due to their unprecedented potential for realizing tailorable integrated devices^10–12^. Then, looking for appropriate optical materials with externally controllable parameters is a leading challenge in modern technology since a plethora of applications is impeded due to the lack of adjustability. Thereby, the tunability of electromagnetic radiation-matter interaction is highly desirable for many applications that rely on the proper variation of their characteristics via particular external factors. Dynamic control can be accomplished by the correct integration of prevailing designs with active media such as liquid crystals, graphene, and phase-transition materials (PTMs)^13–18^. To the best of our knowledge, PTMs can win this competition owing to provide widely tunable capabilities in data storage, sensors, thermochromic applications, optical switches, polarizers, and absorbers since they switch reversibly between two different states in response to external stimuli by applying an electrical, optical or thermal excitation^19–29^. Among the functional PTMs, vanadium dioxide (VO$_2$) as a first-order semiconductor-to-metal transition (SMT) material has attracted considerable attention due to its remarkable electric and optical properties. VO$_2$, first discovered by Morin^30^, experiences the SMT at the critical temperature of T = 340 K, below which it is a semiconductor or an isotropic metal otherwise, under a variety of stimuli such as temperature, stress, and magnetic or electrical field^31–33^. VO$_2$ is a promising choice for applications in thermal responsive devices since its reversible transition from semiconductor to metal state leads to a significant change of its conductivity and optical constant in the rise of the heat process^34^. The SMT of VO$_2$ is accompanied by a striking increase of the infrared (IR) absorption^35^. Particularly, IR transmission and reflection are dramatically affected by the change of temperature while transmission of visible light is nearly invariant during the transition process^36^. Then, based on these properties, VO$_2$ has been extensively used in the development of active and tunable photonic devices like selective absorbers and thermal emitters^4,37–39^.

Diverse features of optical structures involving VO$_2$ have been explored through various reports. An active THz metamaterial made of VO$_2$ cut-wire resonators fabricated on glass substrate was proposed in 2010^40^. Kats et al. proposed a perfect absorber comprising an ultra-thin ($\uplambda /65$) film of VO$_2$ on a sapphire substrate leading to 99.75$\%$ absorption at $\uplambda =11.6~\upmu \text {m}$^41^. Infrared Y-shaped plasmonic antennas, the resonances of which can be tuned or switched on/off by utilizing VO$_2$ PTM, were fabricated in 2013^42^. Song et al. experimentally demonstrated a switchable ultrathin terahertz quarter-wave plate (QWP) by hybridizing metasurfaces with VO$_2$ in which the effective length of the metal resonators in the metasurface alteres through the phase transition of inserted VO$_2$. Then the operating frequency of the QWP becomes tunable and a linearly polarized light is converted into a circularly polarized light at the corresponding operating frequencies^19^. Sanchez et al. exhibited tunable polarizers compatible with silicon photonics for the first time. This tunable transverse electric pass polarizer is based on hybrid VO$_2$/Silicon technology. They can control the rejection of the unwanted polarization via tuning the optical losses across the phase transition of VO$_2$^43^. Lei et al. demonstrated a unique strategy for dynamic broadband absorption in the range from the near to mid IR. This thermally tunable absorber is composed of chromium (Cr) top caps, thermo-chromic VO$_2$ spacers, and a Cr film substrate. They showed that an ultra-broadband absorption over the range of 1627-4696 nm is successfully achieved. Specifically, the 90$\%$ absorption bandwidth changes from 3069 to 632 nm as VO$_2$ transits from a semiconductor to metal state, and the respective average absorptions over the corresponding bands are as high as 93.5$\%$ and 96$\%$^37^. Recently, Shibuya et al. investigated the switching time of micrometer-scale optical modulators consisting of a Si waveguide with a VO$_2$ cladding layer by exploiting the photo-thermal effect. The device exhibites stable optical switching with a high extinction ratio exceeding 16 dB. The dependence of the switching time on the incident light power is studied during the heating and cooling process^44^. Quite recently, Ren et al. introduced a switchable bi-functional metamaterial composed of a hybrid gold-VO$_2$ nanostructure in which the perfect absorption and asymmetric transmission can be thermally switched for circularly polarized near-IR lights^45^.

Photonic crystals (PCs), structures with periodic modulation of refractive index, have become an indispensable technology across the entire field of optical physics because of their capability to control and confine electromagnetic (EM) waves^46^. Moreover, introducing nonlinear elements into PCs is of great scientific interest since it provides new design opportunities, making them highly appropriate to demonstrate lots of phenomena such as optical phase conjugation, third-harmonic generation, self-focusing of light, four-wave mixing, and optical bistability^47–51^. Kerr nonlinearity brings about the dependence of the refractive index on light intensity. Therefore, by integrating Kerr nonlinearity into a PC, one can dynamically control the EM wave propagation. Based on these facts, designing nonlinear PCs has been recognized as one of the hot topics since they can bring us one step closer to the development of beneficial optical devices such as all-optical signal processors and short pulse compressors^52,53^.

In a nutshell, according to the aforesaid clarification, I have been motivated to present an innovative nonlinear one-dimensional (1D) PC including the VO$_2$ defect layer to actively modulate the absorption behavior of the near-IR lights through the phase transition process. First, I show that tailoring the maximum value of absorption is accessible by altering the period number on both sides of the VO$_2$ defect layer. Afterwards, it is demonstrated that a narrow-band, nearly complete absorption of near-IR waves can be obtained thanks to the creation of a defect mode in the structure. The absorption properties of the structure are explored under the phase transition character of VO$_2$. Finally, the influence of the nonlinearity on the absorption is studied. This study offers new opportunities for the control of absorption in 1D nonlinear PCs including a defect layer of PTM. The rest of this paper is organized as follows: the model of the structure and the theoretical calculation are given in Section 2; the numerical analysis and discussion of the spectral characteristics of the structure are presented in Section 3; finally, Section 4 is devoted to the conclusions.

## Theoretical model

Consider a defective 1D PC in the air with the structure of $(AB)^ND(BA)^M$ which is composed of materials *A* and *B* stacked alternately along the z-axis and a defect layer *D*. Notice that the constituent layers of the structure are assumed to be lossless in this paper. Therefore, all the absorption of the structure is due to the VO$_2$ layer. Throughout this work, all layers are supposed to be nonmagnetic ($\mu =1$). The layer of *A* is considered to be a Kerr-type nonlinear material (polydiacetylene 9-BCMU) whose refractive index is written as $n_{A}^{NL}=n_{A}(1+\chi ^{(3)}I/2)$, where $n_A=1.55$ is the linear refractive index, $\chi ^{(3)}=2.5\times 10^{-5}~{\text {cm}}^2/\text {MW}$ is the nonlinear coefficient, and *I* is the electrical field intensity. The layer of B is considered to be isotropic dielectric (TiO$_2$) with the refractive index of $n_B=2.31$. *N* and *M* stand for the number of periods. The thicknesses of layers *A* and *B* satisfy the quarter-wave condition i.e., $n_Ad_A=n_Bd_B=\uplambda _0/4$, where $\uplambda _0$ is chosen to be 800$~\text {nm}$. The layer of *D* denotes a defect layer of VO$_2$ with the thickness $d_D=35$ nm and refractive index $n_D=\sqrt{\varepsilon _D}$. Since VO$_2$ acts like a natural metamaterial including semiconducting and metallic inclusions that are considerably smaller than the working wavelength at and near the phase transition condition, its optical properties could be assessed employing Maxwell-Garnett effective medium theory^54^. Therefore, the VO$_2$ layer is mainly described by its relative permittivity $\varepsilon _D$ which is expressed as a function of *f*, $\omega$ as:

1 $$\begin{aligned} \frac{\varepsilon _D^{\omega }-\varepsilon _{s}^{\omega }}{\varepsilon _D^{\omega }+2\varepsilon _{s}^{\omega }}= f\frac{\varepsilon _{m}^{\omega }-\varepsilon _{s}^{\omega }}{\varepsilon _{m}^{\omega }+2\varepsilon _{s}^{\omega }} \end{aligned}$$

where *f* is a temperature-dependent factor expressing the phase transition of VO$_2$. $\varepsilon _{s}^{\omega }$ and $\varepsilon _{m}^{\omega }$, described in Ref.^55^ in more detail, are the relative permittivities of VO$_2$ in the semiconducting and metallic phases corresponding to $f=0$ and $f=1$, respectively. The temperature dependence of factor *f* is illustrated by Chettiar et al. during the heating and cooling cycles^56^. It is supposed that the transition from the semiconducting to metallic state starts and ends around 335 K and 345 K, respectively, during the heating process. Whereas VO$_2$ experiences a metal-to-semiconductor phase transition when cooled down from 335 K to about 325 K.

Suppose a plane wave be incident from the air upon the interface of the structure at an incident angle $\theta$ with $+z$ direction. All the layers of our structure are parallel to the $x-y$ plane and the *z*-axis is normal to the interfaces of the layers. The absorption spectra of the structure can be analyzed using the well-known transfer matrix method^9,57^ as follows:

2 $$\begin{aligned} A=1-{{\left| \frac{1}{{\chi _{11}}}\right| }^{2}}-{{\left| \frac{{\chi _{21}}}{{\chi _{11}}}\right| }^{2}} \end{aligned}$$

where $\chi _{11}$ and $\chi _{21}$ are the elements (1, 1) and (2, 1) of the total transfer matrix of the proposed structure, respectively. As expressed in the following equation, $\chi$ connects the incident, reflected and transmitted electric (magnetic) fields at the incidence and exit ends for a TE (TM) polarized wave.

3 $$\begin{aligned} \chi ={{\chi }_{in}}\prod \limits _{j=1}^{N}{{{\left( {{\chi }_{A}}{{\chi }_{B}}\right) }_{j}}}{{\chi }_{D}}\prod \limits _{j=1}^{M} {{{\left( {{\chi }_{B}}{{\chi }_{A}}\right) }_{j}}}{\chi }_{out} \end{aligned}$$

The meaning and value of each parameter in Eq. (3) are comprehensively given in Refs.^9,58^.

## Results and discussion

**Figure 1:**
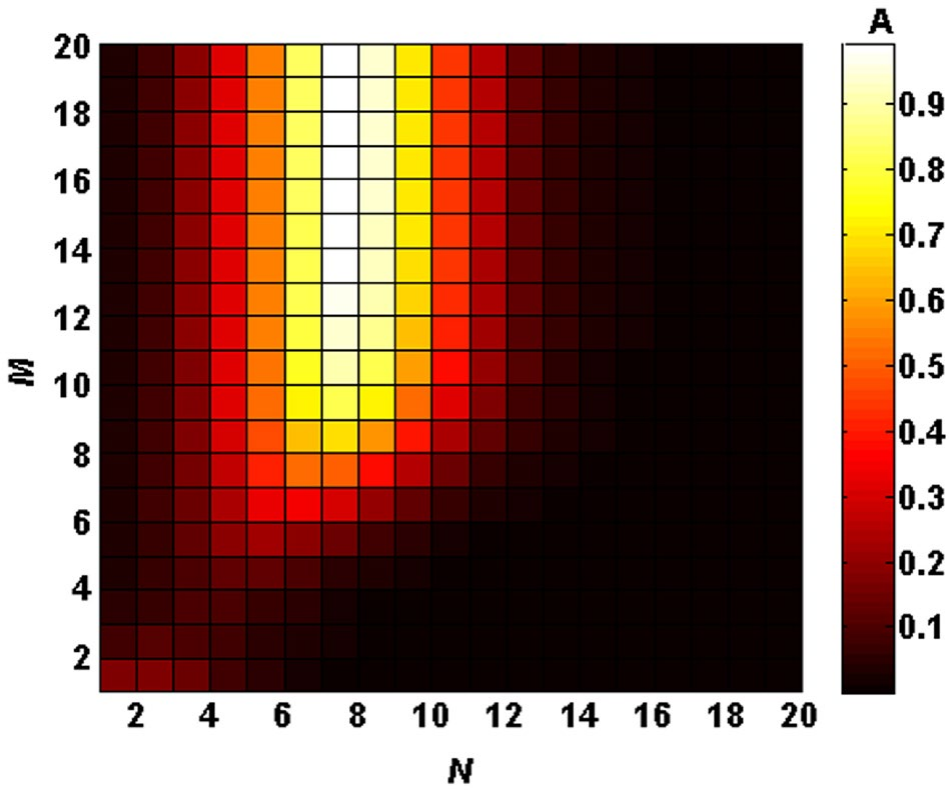
Chart of the absorption peak value of the defective structure $(AB)^ND(BA)^M$ as a function of the period numbers *N* and *M*.

**Figure 2:**
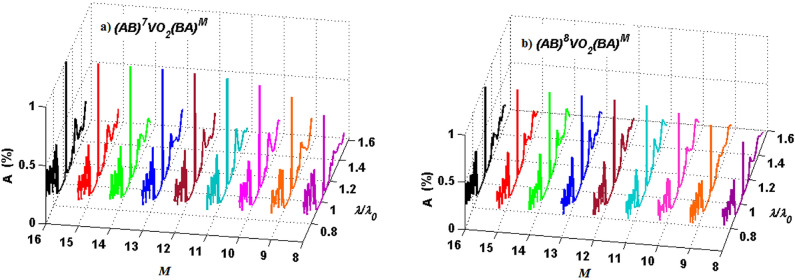
Absorption spectra of the defective structure $(AB)^ND(BA)^M$ as a function of $\uplambda /\uplambda _0$ for (**a**) $N=7$, and (**b**) $N=8$ for different iteration numbers of *M* at normal incidence for semiconducting phase of VO$_2$.

In this section, I numerically evaluate the absorption response of the considered 1D defective PC at the normalized wavelength range of $0.7<\uplambda /\uplambda _0<1.6$. First, I investigate the linear characteristics of the structure provided that all the layers are linear ($\chi ^{(3)}=0$). Since the structure is a defective PC, a defect mode causing the localization of the electric field around the defect layer is expected. So, an enhanced absorption can be obtained. In this regard, I plot Fig. 1 to study the absorption peak value of the structure as a function of the period numbers on both sides of the defect layer at the normal incidence for semiconducting phase of VO$_2$ ($f=0$). Here, white (black) color represents absorption coefficient equal to 1 (0) corresponding to the color legend band next to the figure. As Fig. 1 reveals, there is an optimal period number for the left side of the defect layer, i.e., $N=7$ to maximize the absorption of the structure, while the period number of the right side has negligible or no effect when the period number is increased over $M\ge 16$. Since the absorption value of the structure in the cases of $N=7$ and $N=8$ seems the same, I plot Fig. 2 to compare their absorption spectra distinctively. Figure 2a,b illustrate absorption spectra of the structure $(AB)^ND(BA)^M$ at the normal incidence as a function of right iteration number *M* in two cases (a) $N=7$ and (b) $N=8$. As it is obvious, absorption is considerably increased by increasing *M* up to 16. Furthermore, the peak absorptance of the structure with $N=7$ and $N=8$ are about 99$\%$ and 93$\%$, respectively when $f=0$. These results are in a good agreement with the outcomes of Fig. 1. Then in what follows, *M* is fixed at 16 and *N* is set equal to 7.

**Figure 3:**
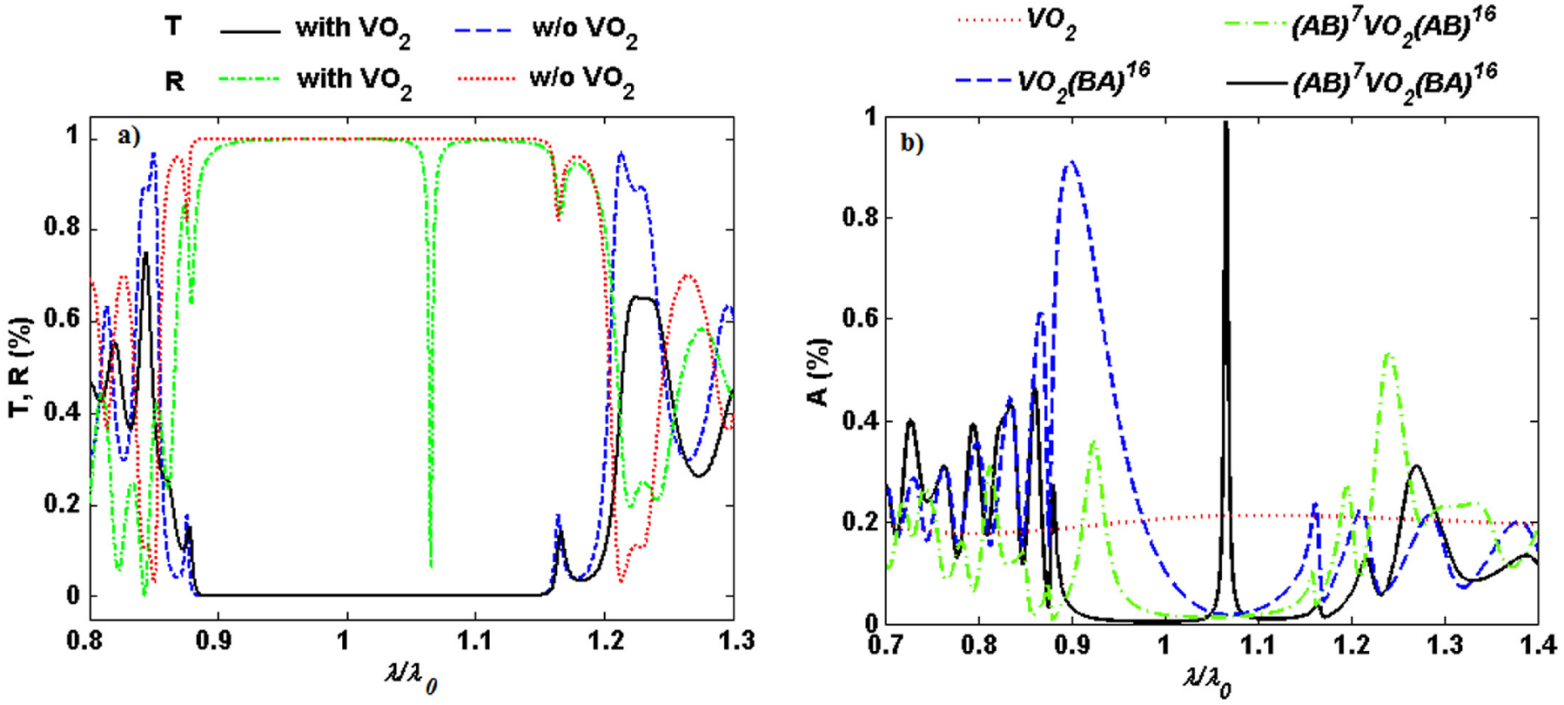
(**a**) Transmission (T) and reflection (R) spectra of the structure $(AB)^7D(BA)^{16}$ with (black solid and green dashed-dotted lines,respectively) and without (blue dashed and red dotted lines, respectively) VO$_2$ as a function of normalized wavelength $\uplambda /\uplambda _0$ under normal incidence. (**b**) comparison of the absorption spectra versus $\uplambda /\uplambda _0$ for bare VO$_2$, VO$_2$ located on top of the periodic structure $(BA)^{16}$, and VO$_2$ used as the defect layer of the 1D asymmetric $(AB)^7D(AB)^{16}$ and symmetric $(AB)^7D(BA)^{16}$ PC (red dotted, blue dashed, green dashed-dotted, and black solid lines, respectively) at normal incidence for semiconducting phase of VO$_2$.

To identify how the inclusion of the VO$_2$ layer affects the optical properties of the structure, the transmission (T) and reflection (R) spectra of the structure with (black solid and green dashed-dotted lines,respectively) and without (blue dashed and red dotted lines, respectively) VO$_2$ under normal incidence are shown in Fig. 3a. The spectra disclose the substantial effect of VO$_2$ on the reflection of the 1D PC at the localized state. For comparison, absorption spectra as a function of normalized wavelength for bare VO$_2$, VO$_2$ located on top of the periodic structure $(BA)^{16}$, and VO$_2$ used as the defect layer of the 1D asymmetric $(AB)^7D(AB)^{16}$ and symmetric $(AB)^7D(BA)^{16}$ PC are depicted in Fig. 3b (red dotted, blue dashed, green dashed-dotted, and black solid lines, respectively). It is clear that although absorption can be enhanced around 0.9$\uplambda _0$ (720 nm) by placing VO$_2$ on top of the periodic structure (VO$_2$ $(BA)^{16}$, blue dashed line),a nearly perfect absorption at 1.066$\uplambda _0$ (853 nm)is achieved only when VO$_2$ is introduced as the defect layer of the 1D symmetric PC ($(AB)^7$VO$_2$ $(BA)^{16}$, black solid line). In addition, absorption of the structure $(AB)^7$VO$_2$ $(BA)^{16}$ at normal incidence is improved about 50-fold with respect to a free standing VO$_2$. The reason of the obtained dramatic rise in the absorption of the defective 1D symmetric PC lies in the fact that magnitude of the electric field increases sharply around the defect layer’ s position. So the symmetric structure $(AB)^7$VO$_2$ $(BA)^{16}$ is selected for the succeeding analysis.

**Figure 4:**
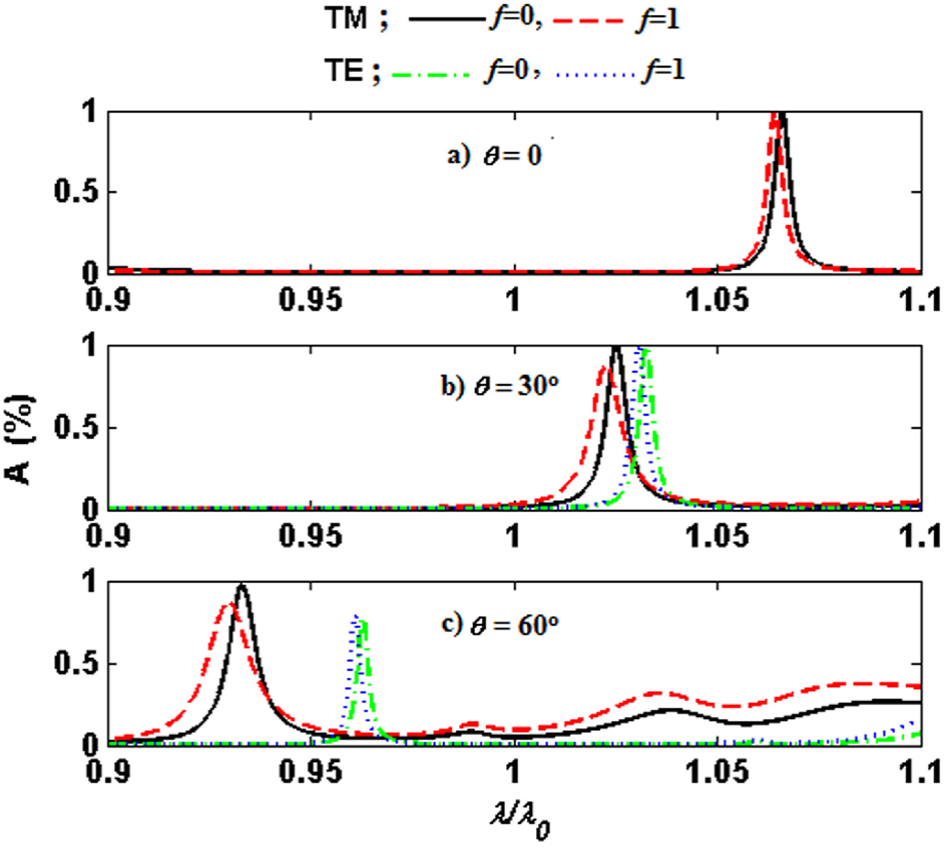
Absorption spectra of the structure $(AB)^7D(BA)^{16}$ versus $\uplambda /\uplambda _0$ for the semiconducting (TM; black solid line, TE; green dashed-dotted line) and metallic (TM; red dashed line, TE; blue dotted line) phases of the VO$_2$ at the incident angles of 0$^{\circ }$, 30$^{\circ }$, and 60$^{\circ }$, respectively.

Next, I will examine the sensitivity of absorption to the temperature-dependent factor *f*. Figure 4a–c show the absorption spectra of the structure as a function of normalized wavelength for different values of filling fraction $f=0$ (black solid line for TM-polarized wave and green dashed-dotted line for TE-polarized one) and $f=1$ (red dashed line for TM-polarized wave and blue dotted line for TE-polarized one) at the incident angles 0$^{\circ }$, 30$^{\circ }$, and 60$^{\circ }$, respectively. As can be observed in Fig. 4a–c, changing the phase of the VO$_2$ layer through increasing temperature to around 340 K may tune over the absorption properties, getting the variation in the resonant peak position and relevant intensity. It should be noted that there is a decrease in the absorption peak with the incident angle deviating from normal incidence, which is more dominant for the TE-polarized wave than TM one. In other words, the structure absorbs the oblique TM-polarized wave more strongly than the TE one, especially at high incident angles. The decrease in absorption stems from the fact that the traveled optical path length through the structure becomes longer as the incidence angle increases. Our results evidently reveal that there is a slight sensitivity to the phase of VO$_2$ at normal incidence in which the maximum absorption of the structure is about 99$\%$ and 98$\%$ at the normalized wavelength of 1.066 $\uplambda _0$ and 1.064 $\uplambda _0$ in the semiconducting and metallic phase, respectively. For both TM and TE-polarized waves, I also find that the transition of VO$_2$ from semiconducting to metallic phase (increasing of factor *f* from 0 to 1) causes the defect mode’s position to shift towards the higher frequencies at oblique incidence 30$^{\circ }$ and 60$^{\circ }$, although it is more manifest for TM ones. Moreover, the absorption peak value remains nearly invariant as the parameter *f* increases from 0 to 1 for TE-polarized wave, while it is decreased for the TM mode. These features in the mentioned structure would have potential applications in designing PC devices where tunable absorption or monochromatic filtering are demanded by adjusting external controlling parameters such as temperature.

**Figure 5:**
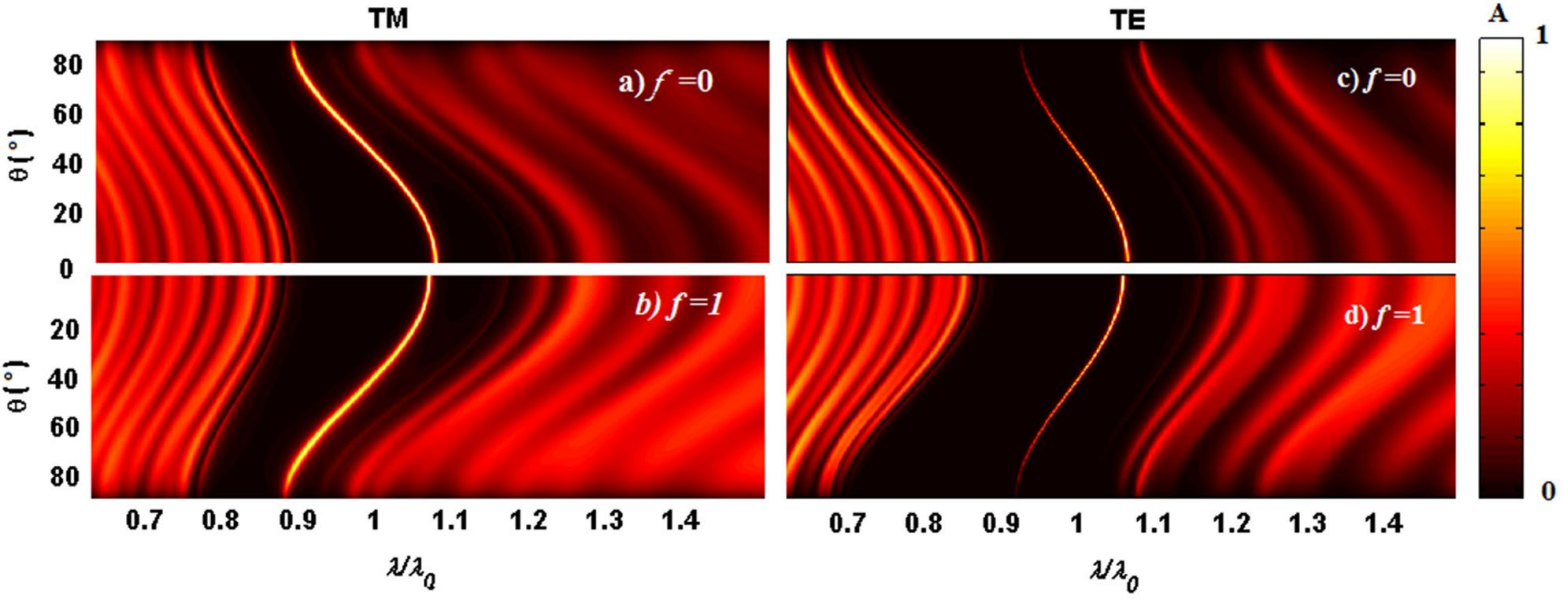
Absorption spectra of the defective structure $(AB)^7D(BA)^{16}$ in the plane of ($\uplambda /\uplambda _0$, $\theta$) for TM polarization (left panels) and TE polarization (right panels) for the semiconducting and metallic phases of the VO$_2$(top and bottom panels, respectively).

To further clarify the angular dependence of the designed structure, the absorption spectra of the structure are plotted in the plane of normalized wavelength and incident angle ($\uplambda /\uplambda _0$, $\theta$) for TM-(right panels) and TE-(left panels) polarized waves in Fig. 5a–d for the semiconducting phase ($f=0$, top panels) and metallic phase of VO2 ($f=1$, bottom panels), also exposing the dynamic tunable nature of the narrow band absorption characteristic via phase changing of VO2 layer. Besides, as one can see the defect mode in all cases shows a blue-shift as the incident angle increases from 0 to 89$^{\circ }$ since the position of absorption peak (correspond to the defect mode) is directly proportional to the cosine of the incident angle according to the resonance condition.

**Figure 6:**
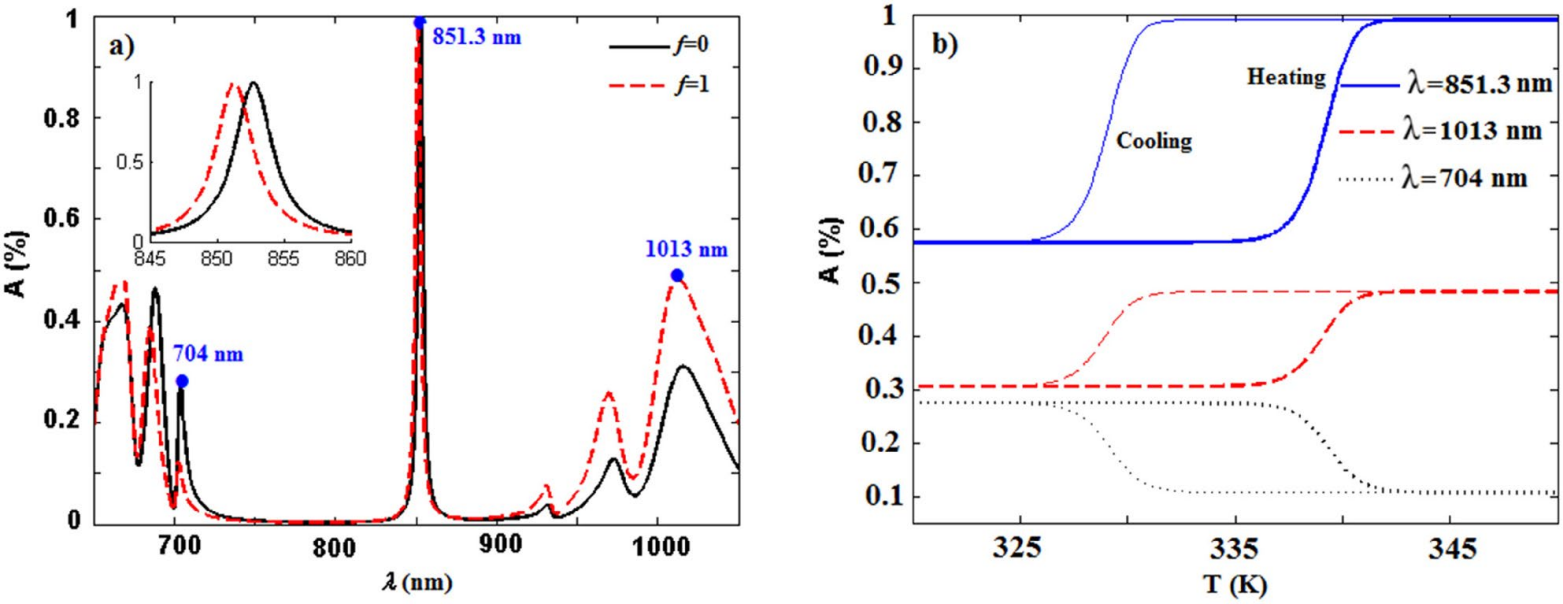
(**a**) Absorption spectra of the structure $(AB)^7$D$(BA)^{16}$ as a function of wavelength $\uplambda$ at normal incidence for the semiconducting (black solid line) and metallic (red dashed line) phases of VO$_2$. The inset shows the absorption of the structure at the wavelength range of 845–860 nm in both cooling and heating modes. (**b**) Temperature-dependent absorption of the structure $(AB)^7$D$(BA)^{16}$ at three different wavelengths $\uplambda =704$, 851.3, and 1013 nm for normal incidence during the heating and cooling cycles.

Since the factor *f* is related to the temperature, the absorption spectrum of the structure thus can be tuned by controlling the temperature. To further demonstrate the tunable behavior, the dependence of absorption on the temperature is also investigated. Figure 6a represents the absorption spectra of the proposed structure $(AB)^7$D$(BA)^{16}$ at normal incidence for both semiconducting and metallic phases of VO$_2$ (solid and dashed lines, respectively). It can be seen from the inset of Fig. 6a that the absorption of the structure at the resonant wavelength of 851.3 nm is about 58$\%$ and 99$\%$ for the semiconducting and metallic states of VO$_2$, respectively. It means that by heating the system (i.e. increasing *f* from 0 to 1), the absorption of the structure increases from 0.58 to 0.99. The absorption rate of the structure at multiple resonant wavelengths is also given in Table 1. The temperature dependence of the absorption at various wavelengths for normal incidence is depicted in Fig. 6b during the heating and cooling process. Here, the thick (thin) lines indicate the heating (cooling) mode. As it is obvious from Fig. 6b, the absorption rate of the resonant wavelength 851.3 nm (1013 nm) dramatically increases from 0.58 to 0.99 (0.3 to 0.48) by raising the temperature from 335 K to 345 K, while there is a reduction in the absorption rate of the wavelength 704 nm in the heating process. On the other hand, it is seen an inverse pattern for absorption of all wavelengths when the temperature declines from 335 K to 325K. Then, the absorption of the structure could be mainly controlled via manipulation of the temperature.

**Figure 7:**
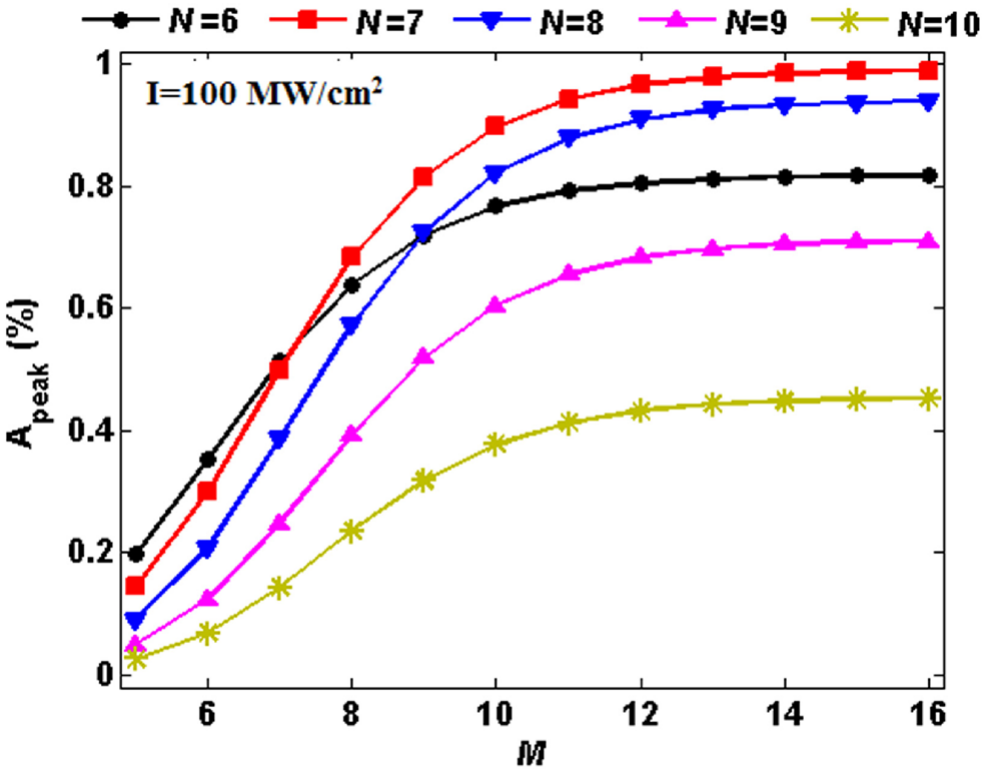
Absorption peak of the nonlinear defective structure $(AB)^ND(BA)^M$ as a function of period number M at normal incidence for different iteration numbers of N. The electrical field intensity and Kerr nonlinearity in all polydiacetylene 9-BCMU layers are assumed to be $I=100$ MW/cm$^2$ and $\chi ^{(3)}=2.5\times 10^{-5}$ cm$^2$/MW, respectively. Here, VO2 is considered in the semiconducting phase.

To illustrate the nonlinear absorption characteristics of the proposed structure, precisely the effect of third-order optical nonlinearity on the resonance peak, I first display the absorption peak value as a function of *M* at normal incidence for different iteration numbers of *N*. Here, VO$_2$ is supposed in the semiconductor phase ($f=0$) and the electrical field intensity is assumed to be $I=100$ MW/cm$^2$. Furthermore, a Kerr nonlinearity with $\chi ^{(3)}=2.5\times 10^{-5}$ cm$^2$/MW is present in all polydiacetylene 9-BCMU layers. As shown in Fig. 7, the intensity of the absorption peak becomes maximum when $N=7$ and $M=16$. So, one may conclude that they are the optimal numbers to reach a near-total absorption (> 99%) like the linear case.

**Figure 8:**
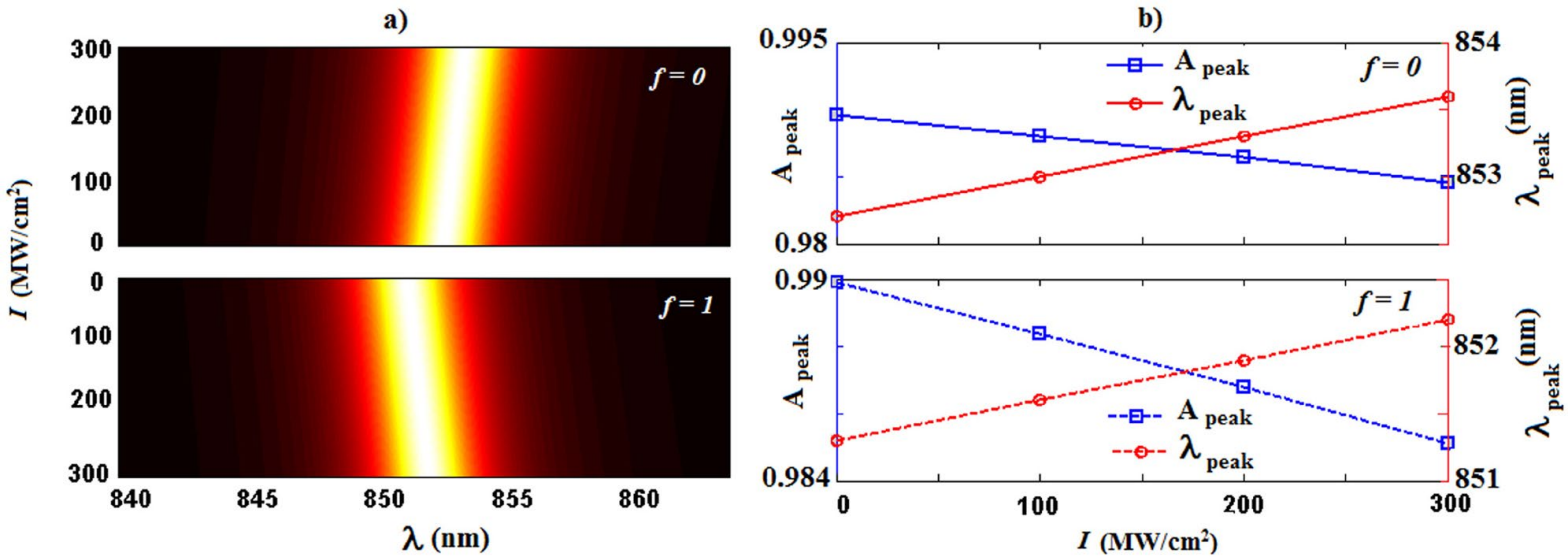
(**a**) Absorption spectra of the nonlinear defective structure $(AB)^7D(BA)^{16}$ at normal incidence. (**b**) Absorption peak values ($A_{peak}$) and resonant peak wavelengths ($\uplambda _{peak}$) versus the field intensity. Here, the top and bottom panels correspond to the semiconducting ($f=0$) and metallic ($f=1$) phases of VO$_2$, respectively.

In this stage, I assess the field intensity dependency of the absorption behavior. Figure 8a demonstrates the tunability of the structure, i.e. the relationship between absorption spectra and the field intensity in the nonlinear regime for $f=0$ (top panel) and $f=1$ (bottom panel) at normal incidence. As the intensity increases from 0 to 300 MW/cm$^2$, the absorption peak shows a red-shift in two metal and semiconductor phases of VO$_2$, whereas a blue-shift in the resonance wavelength occurs while the phase of VO$_2$ changes from semiconducting to metallic state. To get a better insight, the field intensity dependence of absorption peak values and resonant peak wavelength at normal incidence are also illustrated in Fig. 8b. Here, the top and bottom panels correspond to the semiconducting ($f=0$) and metallic ($f=1$) phases of VO$_2$, respectively. It should be noted that when the electric field intensity changes, the refractive index of the nonlinear layers subsequently alters. Then the absorption properties could be dynamically tunable. Besides, it is shown that thermal control of the phase co-existence in the VO$_2$ provides switching of the absorption peak value in addition to the resonant wavelength.

**Table 1:** The absorption rate of the structure $(AB)^7$D$(BA)^{16}$ at various wavelengths for normal incidence during the heating and cooling process.

$\uplambda (nm)$	*f*	A
704	0	0.27
	1	0.10
851.3	0	0.58
	1	0.99
1013	0	0.30
	1	0.48

## Conclusion

In summary, absorption properties of a 1D PC including a defect layer of VO$_2$ are analyzed in the near-infrared frequency region. From the numerical results carried out by the transfer matrix method, it is found that the insertion of a single layer of VO$_2$ in our system can lead to a near-unity and narrow-band absorption due to the localization of the electric field around the defect layer. Besides, it is illustrated that resonant perfect absorption wavelength can be tuned with no significant change in the absorption intensity, by switching the phase of the VO$_2$ layer from semiconducting to metallic states. Moreover, studying the angular dependence of the absorption spectra reveals that the resonant wavelength undergoes a blue-shift as the incident angle increases from 0 to 89$^{\circ }$. I also investigate the tunable absorption behavior of the proposed structure comprising Kerr-type nonlinear layers. It is believed that our structure may have potentially great applications in active nonlinear optoelectronic devices apart from the applications such as sensors, detectors, switches, absorption filters, and so forth.
